# *MYD88* mutations predict unfavorable prognosis in Chronic Lymphocytic Leukemia patients with mutated *IGHV* gene

**DOI:** 10.1038/s41408-017-0014-y

**Published:** 2017-12-15

**Authors:** Shu-Chao Qin, Yi Xia, Yi Miao, Hua-Yuan Zhu, Jia-Zhu Wu, Lei Fan, Jian-Yong Li, Wei Xu, Chun Qiao

**Affiliations:** Department of Hematology, the First Affiliated Hospital of Nanjing Medical University, Jiangsu Province Hospital, Collaborative Innovation Center for Cancer Personalized Medicine, Nanjing, 210029 China

Chronic lymphocytic leukemia (CLL) is the most common leukemia in adults in the Western countries but is relatively rare in East Asia^1^. CLL is a disease of high heterogeneity. The clinical course ranges from indolence to rapid progression to death. Although the Rai and Binet clinical staging systems remain to be the cornerstone for CLL prognosis, the rapidly developed biological and genetic techniques enable the detection of novel prognostic factors.

Mutations in myeloid differentiation primary response gene 88 (*MYD88*) in CLL were first reported in 2011 with a mutation frequency of 9/310 (2.9%)^2^. Subsequent studies found that *MYD88* mutations exist in 2.0–4.4% Caucasian patients with CLL^3–7^. However, subjects of Asia showed a higher *MYD88* mutated rate of 8% as previously reported^8^. The above *MYD88* mutated cases consist mainly of a p. L265P substitution.

CLL patients with *MYD88* mutations were reported to be younger at diagnosis and have longer time to treatment (TTT) and overall survival (OS) than those with wild-type *MYD88*^9^. However, this conclusion was controversial^10^. Initial studies indicated that most *MYD88*-mutated patients belonged to the *IGHV*-mutated group^5,9,11^, which is generally accepted as a molecular sign of favorable prognosis. These studies could be more convincing if taking *IGHV* mutation status and *MYD88* mutations together into prognostic consideration^10^. In the current study, we analyzed *MYD88* mutations exclusively in the *IGHV*-mutated CLL cases to explore its prognostic value.

Two hundred and eighty-four patients with previously untreated CLL at the First Affiliated Hospital of Nanjing Medical University between January 2000 and June 2016 were retrospectively enrolled. All cases were reviewed to confirm the diagnosis according to the 2008 International Workshop in CLL-National Cancer Institute (IWCLL-NCI)^12^. Clinical and biological parameters including absolute lymphocyte count, hemoglobin, platelet, cytogenetic abnormalities, mutation status of *TP53, IGHV, NOTCH1* as well as surface markers of CLL cells were assessed at first presence at our center. The study was approved by the Ethics Committee of the First Affiliated Hospital of Nanjing Medical University with a reference number as 2014-SR-204. Informed consents were provided according to the Declaration of Helsinki.

Mononuclear cells from 281 peripheral blood samples and three bone marrow samples of untreated CLL patients were used for AS-PCR assay. Genomic DNA was extracted using the QIAamp DNA Blood Kits (Qiagen, Düsseldorf, Germany) according to the manufacturer’s recommendation. Two different forward primers (FW5′-GTGCCCATCAGAAGCGCCT-3′ and FM5′-GTGCCCATCAGAAGCGCCC-3′) and one reverse primer (5′-AGGAGGCAGGGCAGAAGTA-3′) were used to amplify the wild-type allele or the *MYD88* L265P mutation allele as previously reported^4^. The sensitivity of AS-PCR was 0.625% in the present study. The Sanger sequencing was performed to confirm the AS-PCR assay and to detect *MYD88* mutations other than L265P. Exon 3–5 was amplified by Sanger sequencing with a forward primer (5′- AGCGACATCCAGTTTGTGC-3′) and a reverse primer (5′- AGGCGAGTCCAGAACCAAG -3′)^8^. Amplified fragments were sequenced with both the forward and reverse primers. Both detecting methods were applied on all samples included in the study.

All statistical analyses were performed by SPSS for Windows (version 19.0; IBM Corporation, Armonk, NY, USA) and Graphpad Prism 6. Fisher’s exact test and the chi-square test were used to determine the correlations between *MYD88* mutations and clinical characteristics. Mann-Whitney U test was applied for comparing mean fluorescence intensity (MFI) as a continuous parameter in *MYD88* mutated and wild-type groups. Time to treatment (TTT) was defined as the time from initial diagnosis to first treatment. OS was defined as the time from diagnosis to death or to the last follow-up. TTT and OS curves were estimated by the Kaplan-Meier method and compared by the log-rank test. The prognostic impact of *MYD88* mutations on TTT and OS was assessed using both univariate and multivariate Cox analysis. All statistical tests were two-sided, and *P* value < 0.05 was considered to be significant.

A total of 284 CLL patients were included in our study. Clinical and biological characteristics are summarized in Table 1. The median proportion of CD19^+^CD5^+^ cells in the samples was 65.3% (range 32.3–98.1%). Using both AS-PCR and Sanger sequencing, we detected *MYD88* mutations (*n* = 25) in 25/284 (8.8%) patients with the hotspot L265P substitution representing 72.0% (18/25) of all mutations. Other detected mutations were all single-nucleotide substitutions including S219C (*n* = 3), V217F (*n* = 2), M232T (*n* = 1) and S243N (*n* = 1).

**Table 1:** Characteristics of the CLL patients according to *MYD88* mutation status

	All (*n* = 284)		MYD88 wild type (*n* = 259)		MYD88 mutated (*n* = 25)		
Characteristic	n*	%	n*	%	n*	%	*P*
Age, *y* (range)	60 (54–69)		60 (54–69)		60 (54–66)		0.512
Male	183	64.4	164	63.3	19	76.0	0.275
Binet C	86	32.1	77	31.3	9	40.9	0.351
*IGHV* mutated	165	59.1	143	56.3	22	88.0	0.002
CD38 ≥ 30%	52	18.8	52	20.6	0	0.0	0.011
ZAP70 ≥ 20%	71	28.7	66	29.1	5	25.0	0.802
*TP53* disruption	62	22.5	58	23.0	4	16.7	0.613
HBV ( + )	62	21.9	54	20.8	8	33.3	0.195
+ 12	42	18.0	40	18.9	2	9.5	0.383
*ATM* deletion	37	17.1	30	15.2	7	36.8	0.026
*NOTCH1*	17	7.5	17	8.1	0	0.0	0.373

*Median and 25th–75th percentiles are reported for continuous variables

Patients with *MYD88* mutations preferentially carried mutated *IGHV* genes (*MYD88* mutated: 22/25 *vs. MYD88* wild-type: 143/254, *P* = 0.001). None of the *MYD88* mutated CLL patients showed CD38 positivity (defined as ≥30%) (*P* = 0.011). Besides, *MYD88* mutated CLL were more frequently ATM-deleted (36.8%, *P* = 0.026). In addition, we observed lower CD200 MFI in *MYD88* mutated CLL patients (*P* < 0.001) within both the overall cohort and CLL patients with mutated *IGHV*. None of the mutated patients had Ig paraproteinemia in our analysis. No difference was observed in the distribution of *TP53* disruptions between *MYD88* wild-type and mutated subjects in the mutated *IGHV*-CLL (referred as M-CLL) (19 vs. 14%, *P* = 0.767).

**Fig. 1:**
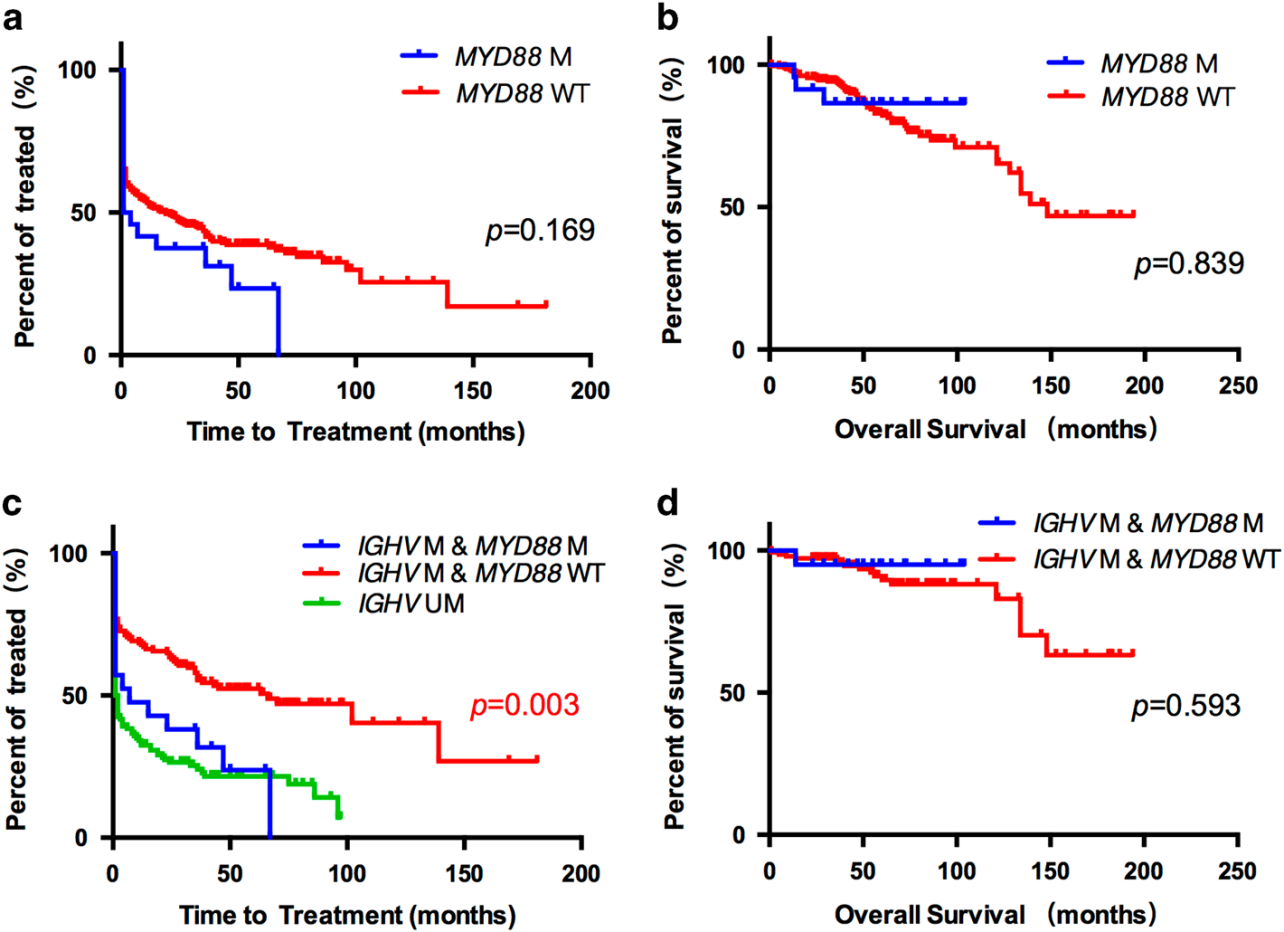
**a** Kaplan-Meier estimates of TTT according to *MYD88* mutation status among all patients. Time to treatment analysis according to *MYD88* mutation status in the CLL patients (*N* = 284). *MYD88* wild-type cases (*MYD88* (-)) are represented by the red line. *MYD88* mutated cases (*MYD88* ( + )) are represented by the blue line. **b** Kaplan-Meier estimates of OS according to *MYD88* mutation status among all patients. Overall survival analysis according to *MYD88* mutation status in the CLL patients (*N* = 284). *MYD88* wild-type cases (*MYD88* (-)) are represented by the red line. *MYD88* mutated cases (*MYD88* ( + )) are represented by the blue line. **c** Kaplan-Meier estimates of TTT according to *MYD88* mutation status and *IGHV* mutation status among M-CLL patients. Time to treatment analysis according to *MYD88* mutation status and *IGHV* mutation status among all CLL patients (*N* = 284). Of the M-CLL cases, *MYD88* wild-type cases (*IGHV* ( + ) *MYD88* (-)) are represented by the red line, while *MYD88* mutated cases (*IGHV* ( + ) *MYD88* ( + )) are represented by the blue line. *IGHV* unmutated cases (*IGHV*(-)) are represented by the green line. **d** Kaplan-Meier estimates of OS according to *MYD88* mutation status among M-CLL patients. Overall survival analysis according to *MYD88* mutation status in the M-CLL patients (*N* = 165). *MYD88* wild-type cases (*IGHV* ( + ) *MYD88* (-)) are represented by the red line. *MYD88* mutated cases (*IGHV* ( + ) *MYD88* ( + )) are represented by the blue line

With a median follow-up of 54.5 months, *MYD88* mutations showed no significant impact on either TTT or OS (Figs. 1a, b). Then we conducted survival analysis in the M-CLL patients. Variables included in the univariate analysis on TTT were: (1) conventional clinical (Binet staging system) factors; (2) widely accepted genetic (*TP53* disruption, defined as *TP53* mutation and/or deletion, *NOTCH1* mutation *ATM* deletion and 12 trisomy) prognostic risk factors; 3) specific protein expression (CD38 and ZAP70). Univariate Cox analysis selected *MYD88* mutation (HR 1.873; 95% CI 1.067-3.287; *P = *0.029), Binet C (HR 3.617; 95% CI 2.278-5.742; *P < *0.001) and *TP53* disruption (HR 1.798; 95% CI 1.090-2.966; *P = *0.022) as risk factors for shorter TTT, and these three parameters went for multivariate analysis in the next step. Multivariate analysis confirmed *MYD88* mutations (HR 2.233; 95% CI 1.233-4.045; *P = *0.008) alongside with Binet C (HR 3.653; 95% CI 2.244-5.944; *P < *0.001) were independently correlated with shorter TTT (Table 2 and Fig. 1c) in M-CLL patients. However, no difference on OS was observed between *MYD88*-mutated and -unmutated cases in the same cohort (*P* = 0.593) (Fig. 1d).

**Table 2:** Univariate and Multivariate analysis for time to treatment in the M-CLL patients

Risk Factors	Univariate analysis			Multivariate analysis		
	HR	95%CI	*P*	HR	95%CI	*P*
*MYD88* mutation	1.873	1.067-3.287	0.029	2.233	1.233-4.045	0.008
Binet C	3.617	2.278-5.742	< 0.001	3.653	2.244-5.944	< 0.001
*TP53* disruption	1.798	1.090-2.966	0.022	1.454	0.861-2.455	0.162
ZAP70 ≥ 30%	1.004	0.616-1.637	0.988	-	-	-
CD38 ≥ 20%	1.139	0.638-2.033	0.659	-	-	-
*NOTCH1* mutation	2.669	0.651-10.946	0.173	-	-	-
+ 12	1.021	0.547-1.908	0.948	-	-	-
*ATM* deletion	1.830	0.886-3.779	0.102	-	-	-

*HR* hazards ratio; 95% CI, 95% confidence interval;

We further analyzed the correlation between *MYD88* mutations and 6 mostly used *IGHV* genes in M-CLL patients. None of the *MYD88* mutated cases used *IGHV*4-34, the most prevalent *IGHV* gene in the M-CLL cohort, (*P* = 0.015) (Table 3), suggesting that *MYD88* mutation might be *IGHV* gene-biased, and that certain antigen exposure might avoid the emergence of *MYD88* mutations in the pathogenesis of CLL.

**Table 3:** The correlation of MYD88 mutation and 6 mostly used IGHV gene in M-CLL patients in China

	All		MYD88-wild	MYD88-mutated	P value
	n	%	n	n	
**VH4-34**					0.015
yes	28	16.7	28	0	
no	140	83.3	117	23	
**VH3-23**					0.484
yes	20	11.9	16	4	
no	148	88.1	129	19	
**VH3-7**					0.484
yes	20	11.9	16	4	
no	148	88.1	129	19	
**VH4-39**					1.000
yes	5	3.0	5	0	
no	163	97.0	140	23	
**VH4-59**					0.526
yes	5	3.0	4	1	
no	163	97.0	141	22	
**VH3-21**					0.448
yes	4	2.4	3	1	
no	164	97.6	142	22	

In this study, we explored the detection method and clinical relevance of *MYD88* mutations in Chinese patients with CLL. We found *MYD88* mutations: (1) occur in 8.8% CLL patients in our center upon diagnosis; (2) cluster with cases harboring mutated *IGHV*; (3) identify a group of patients with poor prognosis among M-CLL; (4) are rare, if not absent, in *IGHV*-4-34 users. The incidence of *MYD88* mutations was 2.0–4.4% in Caucasian CLL patients^3–5^. However, we have detected a higher frequency of 8.8% in our cohort upon diagnosis. The disparities of ethnic groups may explain the difference in frequencies; meanwhile the application of AS-PCR assay in our study indeed improved the detection sensitivity. AS-PCR is previously used in detecting *MYD88* L265P mutations in Waldenstrom macroglobulinemia and diffused large B cell lymphoma^3,13,14^. Our data showed that AS-PCR is capable of detecting samples with a tumor cell load as low as 0.625%, which is far beyond the sensitivity of Sanger sequencing.

The role of *MYD88* mutations in determining the biological features and clinical outcome of CLL patients remains controversial. The initial study indicated that patients with *MYD88* mutations were diagnosed younger and suffered a moreless advanced clinical stage^9^. Contradictory results, however, were observed in that *MYD88* mutations showed no age and stage preference in CLL patients^7,11^, nor does our data do. In the subgroup analysis of M-CLL, we observed *MYD88* mutations predict shorter TTT in this category with favorable outcome. Furthermore, CLL patients with *MYD88* mutations had comparable prognosis with those with unmutated IGHV in our cohort, implying *MYD88* mutations may counteract the survival advantage of mutated *IGHV* gene.

Early research has shown that CLL cells with *MYD88* mutation co-immunoprecipitates with a larger amount of IRAK1&IL-1/TLR signaling pathway, and that activation of the IL-1/TLR pathway promotes proliferation in CLL cells^15^. Furthermore, *MYD88* mutated CLL cells have higher phosphorylation and more DNA-binding activity in NF-κB subunits than CLL cells with wild-type *MYD88*. All these results suggests *MYD88* mutation is a gain-of-function molecular change which may aberrantly activates NF-κB signaling pathway in CLL cells^2,9^ and offers explanation for the unfavorable prognostic impact of *MYD88* mutation on the M-CLL subgroup.

We also found patients with *MYD88* mutations have a relatively lower CD200 MFI compared to the wildtype cases do, consistent with a previous report^16^. Along with the fact that none of the *MYD88* mutated CLL patients expressed positive CD38 in our study, we postulate that this subgroup of CLL patients may have a distinct immunophenotype from CLL without *MYD88* mutations. This will be further explored by targeted RNA sequencing and whole genome sequencing. *MYD88* mutations are mutually exclusive of *IGHV* 4-34 gene usage, which was not shown before to our knowledge. Unlike previously reported, we did not observe a preferable *IGHV* 3-23 gene usage in *MYD88*-mutated cases^10^.

In conclusion, in our cohort of newly diagnosed CLL patients, *MYD88* mutations showed an incidence of 8.8%, including 6.3% on the hotspot missense mutation L265P. *MYD88* mutations predict unfavorable prognosis within the M-CLL subgroup.
